# Variants of the *PPARD* Gene and Their Clinicopathological Significance in Colorectal Cancer

**DOI:** 10.1371/journal.pone.0083952

**Published:** 2013-12-31

**Authors:** Ivana Ticha, Sebastian Gnosa, Annika Lindblom, Tao Liu, Xiao-Feng Sun

**Affiliations:** 1 Division of Oncology, Department of Clinical and Experimental Medicine, Faculty of Health Sciences, County Council of Östergötland, University of Linköping, Linköping, Sweden; 2 Department of Molecular Medicine and Surgery, Karolinska Institutet, Stockholm, Sweden; Sapporo Medical University, Japan

## Abstract

**Background:**

Peroxisome proliferator-activated receptor delta (PPARD) is nuclear hormone receptor involved in colorectal cancer (CRC) differentiation and progression. The purpose of this study was to determine prevalence and spectrum of variants in the *PPARD* gene in CRC, and their contribution to clinicopathological endpoints.

**Methods and Findings:**

Direct sequencing of the *PPARD* gene was performed in 303 primary tumors, in blood samples from 50 patients with ≥3 affected first-degree relatives, 50 patients with 2 affected first-degree relatives, 50 sporadic patients, 360 healthy controls, and in 6 colon cancer cell lines. Mutation analysis revealed 22 different transversions, 7 of them were novel. Three of all variants were somatic (c.548A>G, p.Y183C, c.425-9C>T, and c.628-16G>A). Two missense mutations (p.Y183C and p.R258Q) were pathogenic using *in silico* predictive program. Five recurrent variants were detected in/adjacent to the exons 4 (c.1-87T>C, c.1-67G>A, c.130+3G>A, and c.1-101-8C>T) and exon 7 (c.489T>C). Variant c.489C/C detected in tumors was correlated to worse differentiation (*P* = 0.0397).

**Conclusions:**

We found 7 novel variants among 22 inherited or acquired *PPARD* variants. Somatic and/or missense variants detected in CRC patients are rare but indicate the clinical importance of the *PPARD* gene.

## Introduction

Worldwide, diagnosis of colorectal cancer (CRC) ranks second in females and third in males, and is the fourth most common cause of cancer mortality. Moreover, the incidence rate of CRC is on the rise [1]. The pathogenesis of CRC is complex and still not fully understood. CRC is thought to be a multi-step process implicating an accumulation of genomic aberrations, failure of apoptosis, and abnormalities of multiple signaling pathways [2]. A recent study of Swedish families affected by CRC demonstrates the significant effect of the genetic background of familial CRC patients [3]. Those genes involved in initiation and progression of CRC are limited. Low-penetrant genes may play an important role in colorectal tumorigenesis and need to be identified.

During the last decade, great attention has been given to the investigation of the role of peroxisome proliferator-activated receptor delta (PPARD) in CRC [4]. Previously, Sun’s research group performed mRNA and protein analyses in CRC tissue and cell lines, which suggested an inhibitory role of PPARD in colorectal tumorigenesis [5]–[8]. Harman *et al*. [9] provided also strong evidence that PPARD attenuates colon carcinogenesis, using genetically modified mice models. Specifically, PPARD promotes differentiation and suppresses cell proliferation, which is supported also by others [10]. Furthermore, Sun and co-workers found a correlation between the higher expression of PPARD and favorable survival in rectal cancer patients [6].

This ligand-activated transcription factor belongs to the nuclear hormone receptor superfamily, and is coded by the gene *PPARD* (MIM# 600409) which has nine exons, five of those are coding, and spans around 85 kb on chromosome 6p21.2 [11]. PPARD can be activated by fatty acids and their derivatives, and its expression is relatively high in the gastrointestinal tract compared to other tissues [12]. PPARD has been shown to be involved in regulation of lipid and glucose metabolism and related disorders [13]–[15], and is considered a promising drug target for the treatment of metabolic syndrome diseases [16].

Despite the emerging consensus that PPARD is a key player in CRC, divergent findings complicate the specific role in tumorigenesis. Whether PPARD has a promoting or inhibiting role in colorectal carcinogenesis is still under debate [17], [18]. Genetic variants in the *PPARD* gene might be responsible for these controversial findings and to date the role of *PPARD* variants in CRC has not been investigated. Only a few studies investigated the relationship between polymorphisms in the *PPARD* gene and features of lipid and carbohydrate metabolism [19], [20]. Coding exons 4 to 9 of the *PPARD* gene were sequenced in the large human genome analysis of breast and colorectal cancers [21], [22]. However, *PPARD* was not validated as candidate cancer gene in these analyses. Variant c.489T>C (rs2076167) was enclosed in a search for candidate alleles susceptible for CRC but no correlation with the risk of CRC was found [23].

PPARD has been demonstrated to be involved in CRC development by our group and others. Nevertheless, the significance of the *PPARD* genomic alterations in CRC has not been fully addressed. The goals of the present study were (*i*) to determine the frequency and spectrum of variants in the *PPARD* gene in four different cohorts of CRC patients including 303 tissue samples from CRC, and 150 blood samples from: 50 sporadic CRC patients, 50 patients with 2 affected first-degree relatives, and 50 hereditary patients with ≥3 affected first-degree relatives, and (*ii*) to evaluate potential relationship of variants with clinicopathological variables. Six human colon cancer cell lines, commonly used as *in vivo* colon cancer models in Sun’s laboratory and by others, were included in this study.

## Materials and Methods

### Ethics Statement

This study was approved by the Ethics Committees of University of Linköping, Sweden and Karolinska Institutet, Sweden.

### Patients and Healthy Controls

This study included primary CRC tissue, and, when available, distant normal mucosa from carriers of the *PPARD* variant from 303 patients (group I) diagnosed at the University Hospitals in Linköping and Vrinnevi Hospital in Norrköping. Tissue was collected during primary surgery between 1989 and 2004, and stored −70°C. Blood samples were not available for this cohort. Further, blood samples from unrelated CRC patients: 50 sporadic CRC patients (group II), 50 patients with 2 affected first-degree relatives (group III), 50 hereditary patients with 3 or more affected first-degree relatives (group IV), and 360 non-cancer controls, were examined. The blood samples (group II–III) were obtained between 2004 and 2009 from 14 different hospitals in middle Sweden. To estimate population frequency of the *PPARD* variants, control non-cancer subgroup, comprising blood donors recruited during the year 2010 from Uppsala region of Sweden, were used. Both cases and controls were of European ancestry and from Sweden. Written informed consent from the donor or the next of kin was obtained for use of their samples for research purposes. Characteristics of the patients and controls are shown in Table 1. The tumors with better differentiation included well and moderately differentiated tumors, and worse differentiation included poorly differentiated, mucinous or signet-ring cells carcinoma. Tumor differentiation data could not be obtained for groups II to IV.

**Table 1:** Characteristics of colorectal cancer patients and healthy controls.

Variables	Group I	Group II	Group III	Group IV	Controls
	n (%)	n (%)	n (%)	n (%)	n (%)
**Gender**					
Male	156(51)	30 (60)	28 (56)	29 (58)	164 (46)
Female	147(49)	20 (40)	22 (44)	21 (42)	196 (54)
**Age** a					
Mean (years)	72	74	70	68	57
<Mean	125 (41)	17 (34)	18 (36)	22 (44)	195 (54)
≥Mean	177 (59)	21 (42)	22 (44)	26 (52)	165 (46)
**Location** a					
Colon	172 (57)	17 (34)	23 (46)	24 (48)	–
Rectum	130 (43)	16 (32)	11 (22)	13 (26)	–
**Stage** a					
I	37 (12)	7 (14)	10 (20)	5 (10)	–
II	120 (40)	20 (40)	12 (24)	17 (34)	–
III	96 (32)	13 (26)	15 (30)	18 (36)	–
IV	48 (16)	7 (14)	7 (14)	4 (8)	–
**Differentiation** a,b					
Better	196 (65)	–	–	–	–
Worse	104 (35)	–	–	–	–

^a^ Data not available for some cases;

^b^ Better – well and moderately differentiated tumors, Worse – poorly differentiated, mucinous or signet-ring cell carcinoma; Group I – tumors of CRC patients, Group II – sporadic patients, Group III –patients with 2 affected first-degree relatives, and Group IV – hereditary patients.

### Cell Lines

Mutation analysis was performed also in 6 commonly used colon cancer cell lines. The SW480 and SW620 cell lines were obtained from American Type Culture Collection. SW480 cell line was established from a primary colon adenocarcinoma, and the SW620 from a lymph node metastasis, taken from the same patient one year later [24], [25]. The KM12C, KM12SM and KM12L4a cell lines were kindly provided by Prof. I.J. Fidler (M.D. Anderson Cancer center, Houston, TX) [26], [27]. The KM12C is derived from a patient with stage II colon cancer. The KM12SM is a spontaneous liver metastasis arisen from the injection of KM12C into the cecum of nude mice. KM12L4a, an experimental liver metastasis, is produced by repeated intra-spleen injection and harvesting of the liver metastases in nude mice. The colon cancer cell line HCT-116 was a kind gift from Prof. B Vogelstein (The core cell center, Johns Hopkins University, Baltimore, MD) [28], [29]. The cell lines SW480, SW620, KM12C, KM12SM, and KM12L4a were grown in Eagle’s minimal essential medium (Sigma-Aldrich, St. Louis, MO) and the cell line HCT-116 in McCoys5A (Sigma Aldrich) supplemented with 10% heat inactivated fetal bovine serum albumin (GIBCO, Invitrogen, Paisley, UK), 0.5% L-glutamine (GIBCO), 1% of a penicillin and streptomycin cocktail (GIBCO) at 37°C and 5% CO_2_ in humidified incubator. For the KM12 cells 2% vitamin solution (GIBCO) was added. The cells were harvested at 80% confluence.

### Isolation of Nucleic Acids from Tissues, Blood and Cell Lines

DNA was isolated from fresh frozen tumor tissue, cell lines and whole peripheral blood using standard procedures implementing Wizard genomic DNA Purification System (Promega, Madison, WI) or DNeasy Blood & Tissue Kit (Qiagen, Hilden, Germany). Total RNA from particular tissue samples and cultured cells was isolated by using RNeasy Mini Kit (Qiagen) according to the manufacturer's instructions.

### Mutation Analysis

The coding region of the *PPARD* gene was screened by PCR and direct DNA Sanger sequencing in 203 tumors, 150 blood samples of CRC patients (group II–IV), and 6 cell lines. Because of time and cost efficiency, additional 100 tumor samples as well as controls were screened in two most frequently altered regions spanning exons 4 and 7. Adenine of translation initiation codon is 102 base of exon 4. Therefore the exons 4 to 9 and adjacent intronic sequences of the *PPARD* gene were amplified using FastStart High Fidelity PCR System (Roche Applied Science, Germany) according to the manufacturer’s instructions. BigDye Terminator *v*3.1 Ready Reaction Mix (Applied Biosystems, Foster City) was used for sequencing reaction and separation was performed on ABI 3500 genetic analyzer (Applied Biosystems). The collected data were analyzed by using Sequence analyzer software (Applied Biosystems). Designed primers used for amplification and sequencing analysis are shown in Table S1. Each mutation or suspicious fragments were verified by another independent PCR amplification and sequence analysis.

**Figure 1:**
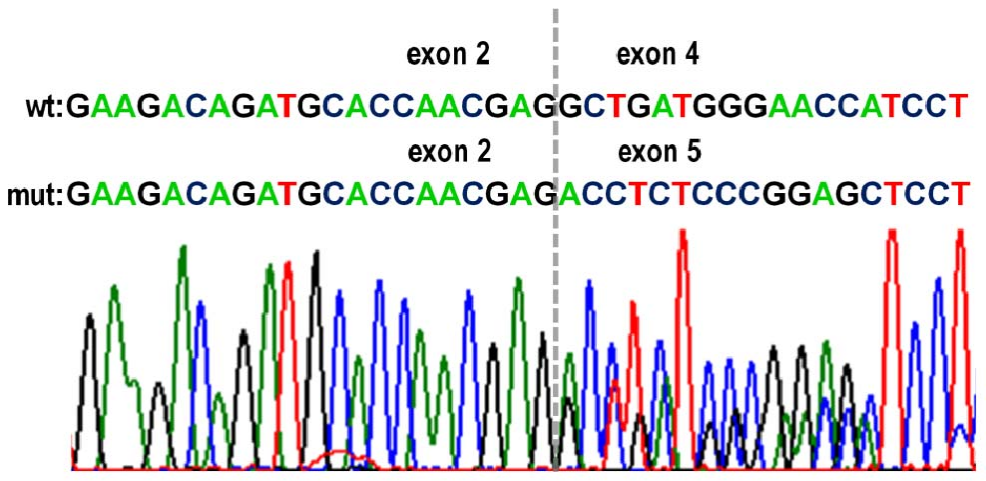
Verification of the impact of germline variants c.130+3G>A and c.1-101-8C>T on posttranscriptional splicing. Representative sequencing analysis of cDNA isolated from normal mucosa from carrier of variants c.130+3G>A (intron 4) and c.1-101-8C>T (intron 3) shows missing of exon 4; wt – wild-type sequence, mut – mutated sequence. Exon junction is indicated by *dashed line*. Exon 3 was absent in both wild type and mutated allele.

Reverse transcriptase-PCR analysis using High Capacity cDNA Reverse Transcription Kit (Applied Biosystems) was performed according to the manufacturer’s instructions in 3 cases carrying variants c.130+3G>A and c.1-101-8C>T with available RNA from corresponding tumor and normal tissue. An 856 bp cDNA fragment bordered by primers RP_01/02F 5′-CAGTGTTGTACAGTGTTTTG-3′ crossing junction of exons 1 and 2, and PRD_08R 5′-TCTGCCTGCCACAATGTCTC-3′ situated in exon 8 was PCR amplified and directly sequenced (Fig. 1). The same cDNA fragment was sequenced in SW480, SW620, KM12C, KM12SM, and KM12L4a cell lines.

### Nomenclature of Mutations

Mutations were described according to the nomenclature system recommended by the Human Genome Variation Society (HGVS) [30]. Designation of the genomic alterations in the *PPARD* gene is based on the GenBank reference sequences NG_012345.1 and NM_001171818.1. Mutations which were not found in the literature, the Single Nucleotide Polymorphism Database (dbSNP, http://www.ncbi.nlm.nih.gov/SNP/, accessed on August 8, 2013) [31], or in the Catalogue of Somatic Mutations in Cancer (COSMIC, http://www.sanger.ac.uk/cosmic, accessed on August 8, 2013 [32], were considered as novel. There is inconsistency in the description of the variants c.1-87C>T (rs2016520) in 5′-untranslated region (UTR) of exon 4, and synonymous substitution c.489C>T (rs2076167; p.N163) in exon 7. According to the GenBank reference sequence (NCBI) the wild type allele is C, but according the genotyping in the control group (Table 2) the C-allele is minor for both variants. It is in concordance with two other studies that performed genotyping including these two polymorphisms in larger cohorts [15], [19]. Of note, Skogsberg *et al*. [15] named variant c.1-87T>C as +294T/C. Hereby, we name these variants c.1-87T>C and c.489T>C.

**Table 2:** Frequency of recurrent *PPARD* variants in colorectal cancer patients and healthy controls.

Exon/intron	Nucleotide change^a^	Group I	Group II	Group III	Group IV	Controls
		n = 303 (%)	n = 50 (%)	n = 50 (%)	n = 50 (%)	n = 360 (%)
**7**	**c.489**					
	T/T	220 (72.6)	40 (80)	33 (66)	40 (80)	270 (75)
	T/C	72 (23.8)	8 (16)	16 (32)	9 (18)	83 (23)
	C/C	11 (3.6)	2 (4)	1 (2)	1 (2)	7 (2)
**4 (5′UTR)**	**c.1-87**					
	T/T	231 (76.2)	36 (72)	35 (70)	41 (82)	269 (75)
	T/C	65 (21.5)	12 (24)	15 (30)	9 (18)	83 (23)
	C/C	7 (2.3)	2 (4)	–	–	8 (2)
**4 (5′UTR)**	**c.1-67**					
	G/G	287 (94.7)	49 (98)	47 (94)	49 (98)	351 (97.5)
	G/A	15 (5)	1 (2)	3 (6)	1 (2)	9 (2.5)
	A/A	1 (0.3)	–	–	–	–
**i3+ i4**	**c.1-101-8C>T+c.130+3G>A**	12 (4)	–	5 (10)	1 (2)	9 (2.5)

^a^ GenBank reference sequence are NG_012345.1 and NM_001171818.1:+1 corresponds to the A of the ATG translation initiation codon; Group I – CRC tumors, Group II – sporadic patients, Group III – patients with 2 affected first-degree relatives, and Group IV – hereditary patients, UTR – untranslated region.

### *In silico* Prediction Tools to Assess the Impact of Detected Missense Variants

For evaluation of functional importance of detected missense variants we employed several widely used *in silico* prediction programs. These programs suggest the possible interference with the biological function and stability of protein: SIFT as a part of commercial Alamut 2.0 program (Interactive Biosoftware, Roven, France), PolyPhen-2 (http://genetics.bwh.harvard.edu/pph2/), PROVEAN (http://provean.jcvi.org/index.php), Align GVGD (http://agvgd.iarc.fr), Mutation Taster (http://www.mutationtaster.org), MUpro (http://mupro.proteomics.ics.uci.edu).

### Statistical Analyses

Analyses were performed by using the STATISTICA 10 (SatSoft, Tulsa, OK). The chi-square test was applied to determine the relationship between recurrent *PPARD* variants and clinicopathological variables, to evaluate the differences in alterations frequencies between groups II–IV and controls, and to test distribution of genotypes in controls for a departure from Hardy-Weinberg equilibrium. Cox’s Proportional Hazard Model was used to test the relationship between PPARD variants and the patient survival, and the Kaplan-Meier method was used for survival curves. All tests were two sided, and a *P*-value less than 0.05 was considered as significant.

## Results

### Variants of the *PPARD* Gene in CRC Patients, Healthy Controls, and Cell Lines

Direct DNA sequence analysis of the *PPARD* gene demonstrates 22 different single nucleotide variants, of those 5 were recurrent and 7 were novel variants (Table 2 and 3). The frequency of five recurrent *PPARD* variants in diverse CRC patient cohorts, healthy controls, and cell lines are shown in Table 2. Genotypic distributions of hereditary recurrent single nucleotide variants were consistent with Hardy-Weinberg equilibrium (data not shown). All detected variants were transitions (4 missense, 3 silent, and 15 noncoding variants). To evaluate the predicted effects of missense variants on protein function, six *in silico* prediction tools were used. In fact, somatic mutation p.Y183C, and mutation p.R258Q were categorized as deleterious (Table 4).

**Table 3:** Description of the *PPARD* variants detected in colorectal cancer patients, healthy controls and cell lines.

Exon/intron	Mutation designation, gDNA^a^, cDNA*^b^*	Predictedmutation effect	References*^c^*	Tumortissue	Normaltissue	Bloodpatients/controls	Cell lines
**i3**	**g.73422C>T**	**co-occurs with**	**rs9658132**	**yes**	**yes**	**yes**	**no**
	**c.1-101-8C>T**	**c.130+3 G>A**					
i3	g.73427C>T	p.?	rs9658133	yes	NA	yes	no
	c.1-101-3C>T						
**4**	**g.73444T>C**	**p.?**	**rs2016520**	**yes**	**yes**	**yes**	**yes** ***^d^***
**(5′UTR)**	**c.1-87 T>C**	**p.?**	**rs2016520**	**yes**	**yes**	**yes**	**yes** ***^d^***
**4**	**g.73464G>A**	**p.?**	**rs9658134**	**yes**	**yes**	**yes**	**no**
**(5′UTR)**	**c.1-67 G>A**						
4	g.73619A>G	p.N30S	rs149040923	yes	NA	no	no
	c.89A>G						
**i4**	**g.73663G>A**	**alternative transcript?**	**rs9658135**	**yes**	**yes**	**yes**	**no**
	**c.130+3G>A**	**(** **Figure 1** **)**					
i4	g.73691C>T	p.?	novel	no	NA	no/yes	no
	c.130+31C>T						
i5	g.82764T>C	p.?	novel	no	NA	no	yes*^e^*
	c.285+40T>C						
i6	g.84432G>A	p.?	novel	yes	NA	no	yes*^f^*
	c.424+31G>A						
i6	g.86380C>T	p.?	novel	yes	no	no	no
	c.425-9C>T						
i6	g.86345G>C	p.?	rs199561824	yes	NA	no	no
	c.425-44G>C						
**7**	**g.86453T>C**	**None**	**rs2076167**	**yes**	**yes**	**yes**	**yes** ***^d^***
	**c.489T>C**	**(p.N163)**					
7	g.86506A>G	p.H181R	novel	yes	NA	no	no
	c.542A>G						
7	g.86512A>G	p.Y183C	COSMIC	yes	no	no	no
	c.548A>G						
7	g.86588G>A	None	rs138479838	yes	yes	no/yes	no
	c.624G>A	(p.T208)					
i7	g.86628G>A	p.?	novel	no	NA	yes/no	no
	c.627+37G>A						
i7	g.86756 G>A	p.?	rs9658162	yes	no	no	no
	c.628-16G>A						
8	g.86917G>A	p.R258Q	novel	no	no	no	yes*^g^*
	c.773G>A						
8	g.87035C>T	None	rs77124831	yes	NA	no	no
	c.891C>T	(p.I297)					
i8	g.87244G>A	p.?	rs180784946	yes	NA	no	yes*^h^*
	c.1078+22G>A						
i8	g.88255C>T	p.?	rs9658165	no	NA	yes/no	yes*^d^*
	c.1079-20C>T						
9	g.88644G>A	p.?	rs201923617	yes	yes	no	no
(3′UTR)	c.1326+122G>A						

^a^ GenBank reference sequence NG_012345.1; ***^b^*** GenBank reference sequence NM_001171818.1: +1 corresponds to the A of the ATG translation initiation codon; ***^c^*** rs# is the reference number from the Single Nucleotide Polymorphism Database (dbSNP); ***^d^*** KM12C, KM12SM and KM12L4a cells; ***^e^*** HCT116 cells; ***^f^*** SW480 and SW620 cells; ***^g^*** SW480 cells; ***^h^*** KM12C cells; COSMIC – the Catalogue of Somatic Mutations in Cancer; common variants are indicated in *bold*; somatic variants are *underlined*; NA – not analyzed/corresponding normal tissue is not available; UTR – untranslated region.

**Table 4:** Prediction of pathogenicity of missense *PPARD* variants.

Exon	Mutationdesignation gDNA^a^,cDNA*^b^*	Predicted mutation effect	SIFT (within Alamut)	PROVEAN	Polyphen-2	MUpro	GVGD*^c^* ^/*d*^	Mutation Taster
4	g.73619A>G, c.89A>G	p.N30S	tolerated	neutral	benign	decrease stability	C0/C0	polymorphism
7	g.86506A>G, c.542A>G	p.H181R	tolerated	neutral	benign	increasestability	C15/C0	disease causing
7	g.86512A>G, c.548A>G	p.Y183C	deleterious	deleterious	probablydamaging	decrease stability	C65/C15	disease causing
8	g.86917G>A, c.773G>A	p.R258Q	deleterious	neutral	probablydamaging	decrease stability	C35/C35	disease causing

^a^ GenBank reference sequence NG_012345.1; ***^b^*** GenBank reference sequence NM_001171818.1: +1 corresponds to the A of the ATG translation initiation codon; ^c^ classifiers C65, C55, C 45, C35, C25, C15, C0 indicate variants most likely (C65) to interfere with function to least likely (C0), for alignment were used following sequences: Homo sapiens, Procavia capensis (rock hyrax), Cavia porellus (guinea pig), mus musculus (mouse), rattus norvegicus (rat), monodelphis domestica (opossum), macropus eugenii (wallaby), Gorilla gorilla (monkey), Gallus gallus (chicken), Orychtolagus cuniculus (rabbit), Taeniopygia guttata (bird); ^d^ the depth of alignment is extended to Xenopus tropicallis (frog), and Danio rerio (zebrafish).

### Recurrent *PPARD* Variant in Relation to Clinicopathological Variables

Association of recurrent variants with clinicopathological characteristics, including gender, age, tumor location, stage and differentiation, was investigated. Variant c.489C/C was significantly related to worse differentiation compare to T/C and T/T genotypes (*P* = 0.0397, Table 5). The clinicopathological data of the patients with detected variant c.489C/C are shown in Table S3.

**Table 5:** *PPARD* variant c.489C/C is related to the worse differentiation in colorectal cancer.

Differentiation^a^	Better	Worse
c.489 genotype	n (%)	n (%)
C/C	4 (36)	7 (64)
T/T+T/C	192 (66)	97 (34)

^a^ Better – well and moderately differentiated tumors, Worse – poorly differentiated, mucinous or signet-ring cell carcinoma.

There was no significant relationship of recurrent *PPARD* variants with other clinicopathological variables including gender, age, tumor location and stage (*P*>0.05, data not shown).

### Characterization of Recurrent *PPARD* Variants

In total, 196 variants in 106 (35%) patients out of group I, 28 variants in 17 (34%) patients out of group II, 45 variants in 22 (44%) patients out of group III, and 22 variants in 11 (22%) patients out of group IV were detected. Five germline recurrent alterations (Table 2), four located in or adjacent to exon 4 (c.1-87T>C, c.1-67G>A, and joint variants c.130+3G>A and c.1-101-8C>T) and one in exon 7 (c.489T>C) accounted for vast majority of all identified variants, and often occurred together without a strict pattern of variant combination. Majority of carriers of at least one minor C-allele at the position c.1-87 at 5′-UTR were also carriers of at least one minor C-allele at the position c.489. Carriers of the variant c.1-67G>A at 5′-UTR of exon 4 carried also another variant, either c.489T/C or c.489C/C, except of one case (group II) with the variants c.1-101-8C>T and c.130+3G>A. The germline intronic variants c.1-101-8C>T and c.130+3G>A, located eight base pairs upstream and 3 base pairs downstream of exon 4, respectively, always occurred together. All patients and controls with these variants were carriers of heterozygous variant c.489T/C, except two patients (group II) – one who carried variants c.489C/C and c.1-67G/A, and one carrier of variant c.1-67G/A.

Mutation analyses in blood samples showed that the amount of the patients carrying inherited alterations were lower in group IV compare with groups II and III combined (*P*  = 0.037), while no difference was observed between group II and III (*P*  = 0.305; Table 2). The frequency of recurrent variants in exons 4 and 7 did not differ significantly between control population and subgroups II, III or IV, except joint variants c.1-101-8C>T and c.130+3G>A, that were found in 10% of patients in group III compared with 2.5% in controls (*P*  = 0.003; Table 2).

Joint variants c.1-101-8C>T and c.130+3G>A are in the vicinity of consensus splice sites and have potential to alter posttranscriptional splicing. To evaluate the impact of these variants, RNAs from tumor and normal tissue of 3 affected individuals and unaffected control were extracted and reverse transcription was performed. Sequence analysis of PCR fragment, comprising region from the end of exon 1 to exon 8 of cDNA, revealed skipping of exon 4 (Fig. 1). All analyzed samples were lacking exon 3. The sequencing of the same cDNA fragment in cell lines also revealed missing of exon 3 (data not shown). Five known alternative transcript variants of PPARD have been described (http://www.ncbi.nlm.nih.gov, Gene ID: 5467) and only one of these variants contains exon 3 in mRNA sequence. Alternative transcript number 4 with absence of exons 3 and 4 has alternative start codon in exon 2 and express protein isoform 3, which differs in N-terminus because of lacking a part of 5′-coding sequence. Other transcript variants have start codon in exon 4.

**Figure 2:**
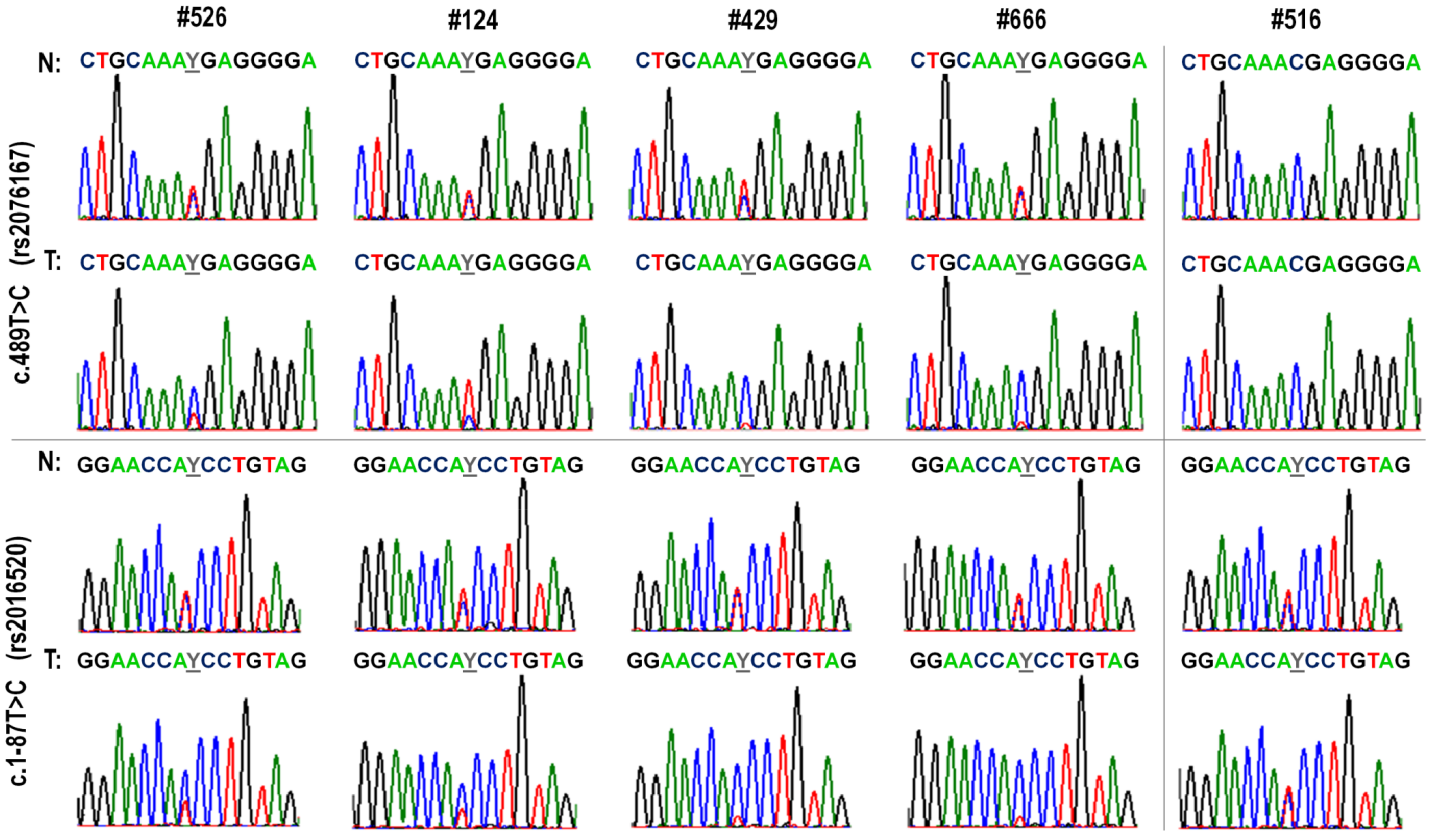
Different sequence profiles in representative matched DNA samples of normal and tumor tissue in polymorphic sites and c.489 (rs2076167) and c.1-87 (rs2016520). N – normal tissue, T – tumor tissue; #516 is example of sequence analysis with the same pattern in normal and tumor tissue.

Analysis in random 20 paired samples (samples that were heterozygotes or homozygotes of mutated variant in at least one of the polymorphic sites c.1-87 or c.489) revealed equal heterozygosity in the polymorphic sites c.1-87T/C and c.489T/C in 4 normal tissues, while in matched tumor DNA samples different proportion of both alleles was observed repeatedly (Figure 2).

### Characterization of Novel and/or Somatic *PPARD* Variants

Seven novel variants were detected in patients, controls and/or in cell lines (Table 3). Out of these, three variants were missense and 6 located in introns. One of these novel intronic variants was somatic, one was found in control group, and one in HCT-116 cell line. All these variants were heterozygous and detected only once. Besides novel variants, another 2 sporadic and 8 rare variants were detected. Characteristics of the patients with detected rare variant are reported in Table S2.

Interestingly, two of three detected somatic variants, c.425-9C>T (seven base pairs from the 3′ consensus splice site) and c.548A>G (in exon 7, leading to the change of highly conserved amino acid tyrosine to cysteine at the codon 183, and occurred together with joint variants c.1-101-8C>T and c.130+3G>A), were detected neither in two other DNA samples extracted from spatially different parts of tumor nor in the corresponding normal mucosa.

The missense mutation c.773G>A (p.R258Q) in exon 8 was detected in SW480 (cell line derived from colon adenocarcinoma) but not in SW620 cells (cell line derived from lymph node metastasis). This variant occurs in a ligand binding domain and leads to the change of highly conserved amino acid arginine to glutamine.

Both missense variants c.548A>G (p.Y183C) and c.773G>A (p.R258Q) were suggested to be pathogenic using *in silico* predictive programs (Table 4).

## Discussion

The present study shows for the first time sequence analysis of the *PPARD* gene in diverse groups of CRC patients, healthy controls, and colon cancer cell line models. We detected 5 recurrent and 17 rare variants, whereof 7 were reported for the first time in this study. Two or more *PPARD* variants, mostly recurrent alterations, occurred together in majority of patients with detected variant. Two out of four detected missense variants (p.Y183C and p.R258Q) were classified by *in silico* prediction programs as likely pathogenic.

Even more interestingly, analyses in a cohort of 303 CRC patients (group I) revealed that the carriers of recurrent variant c.489C/C had worse differentiated tumor compared with the c.489C/T and c.489T/T. However, it is not clear if this variant drives development of tumor, or if it is just a passenger mutation without direct effect on the fitness of the tumor cells.

It has been proposed that cancers of the proximal and distal colon may be two distinct cancer types with different genetic and environmental risk factors, and there were described genetic differences between the cancers that arise in the proximal and the distal colon [33]. Nevertheless, we did not reveal significant differences in distribution of alterations between colon and rectal carcinomas, neither between left and right sided tumors.

Further, three somatic *PPARD* variants, c.425-9C>T, c.548A>G (p.Y183C), and c.628-16G>A, were detected in three sporadic colon tumors (group I). We observed a heterogeneous somatic variant which was not detectable in every sequenced tumor part. The variant c.548A>G has been announced in the COSMIC database of somatic mutations in one patient affected by squamous cell carcinoma [32]. Up to date, the COSMIC contains 40 different *PPARD* somatic variants, scattered in the whole gene, detected in 43 unique patients. Out of these, 3 silent and 7 missense variants were found in 10 mucinous colon adenocarcinomas, and one missense variant in rectal tumor sample. Somatic *PPARD* variants were described also in lung, breast, ovary, endometria, liver, and prostate tumor, and neuroblastoma. The presence of somatic variants in CRC tumors supports the relevance of *PPARD* in CRC tumorigenesis.

Screening in the patient blood samples revealed similar frequency of the recurrent variants located in, or adjacent to, exons 4 or 7, among the patient groups II, III and IV compared with the control group, except of a higher prevalence of the joint variants c.1-101-8C>T and c.130+3G>A in group III. Interestingly, we observed the highest frequency of germline variants in the low risk population of patients with two affected first-degree relatives (group III) whereas the lowest frequency was observed in high risk group of hereditary CRC patients (group IV). However, any substantial interpretation of the data is limited by small sample sets and by little, even though statistically significant, differences. Further analyses of larger series of patients are desired for accurate comparative analyses.

We found novel missense mutation c.773G>A (p.R258Q) in the primary colon cancer cell line SW480 and not in lymph node metastatic cell line SW620. A unique feature of SW480 and SW620 cell lines is that they are derived from primary and secondary tumors resected from the same patient. Another variant, c.1078+22G>A, is present only in primary tumor derived cell lines KM12C but not in the experimental metastatic cell lines KM12SM and KM12L4a. Distinct mutation status in primary tumor cell lines and metastatic cell lines, as well as detection of somatic variants only in a part of the tumor (c.425-9C>T and c.548A>G) are in concordance with the model of clonal evolution of cancer and intratumor genetic heterogeneity [34], [35]. Spatial distribution of subclones with different genomic aberrations within tumor and metastasis was described recently [36]. The occurrence of the *PPARD* mutation in primary cell lines but not in metastatic cell line may be also explained by deletion in *PPARD* loci during tumor and metastasis formation. Moreover, DNA sequence analysis in the matched samples (group I) showed in several cases different proportion of alleles in two polymorphic sites, located in exon 4 and 7, when compared tumor with normal tissue. Considering the fact that the *PPARD* gene is located in the human leukocyte antigen (HLA) complex on chromosomal region 6p, which is frequently affected by deletions and rearrangements in human cancers [37], [38], our observation may suggest loss of heterozygosity in the *PPARD* loci in tumor cell subclones during tumor development.

Of note, vast majority of detected alterations are transcriptionally silent, either located in 5′- or 3′-UTRs or in introns. Several lines of evidence suggest that any nucleotide variant (intronic, nonsense, missense, synonymous) may be considered as potentially deleterious because it may alter normal pre-mRNA splicing *via* changes in consensus splicing sequences, creation of new cryptic sequences, affection of translational rate, or changes of mRNA or protein stability [39]–[41]. Such previously neglected variants could be susceptible for hereditary diseases. Unfortunately, in our study it was impossible to test all variants on mRNA level due to the lack of the particular tissue or blood samples for RNA isolation. Nevertheless, sequence analysis of cDNA in carriers of inherited joint variants c.130+3G>A and c.1-101-8C>T revealed skipping of exon 4, which may lead to translation of the PPARD isoform with changed N-terminus of protein compared with wild type form. The *PPARD* variants at 5′-UTR may be of special interest, because of the hypothesized regulatory role of mRNA expression. However, in the cohort of 303 CRC patients, we did not find an association between recurrent variants in 5′-UTR (c.1-87C>T and c.1-67G>A) and clinicopathological variables.

We would like to strengthen the importance of characterization of the genetic background of cell lines used as model systems. For example, two laboratories showed that PPARD protein expression was dependent on APC/*beta*-catenin pathway [42], [43]. Their conclusion was based on observation that relative expression of PPARD was either similar or lower in SW480 cells that have a mutant *APC* gene and a wild-type *beta*-catenin gene. In the present study we detected missense *PPARD* mutation (p.R258Q) in SW480 cells that can influence the interpretation of those results and indicates limited usage of SW480 cell line for the *in vivo* studies. Further, comprehensive analysis of the *PPARD* gene could be helpful in drug design, since PPARD provides an attractive target for therapeutic intervention so far in patients with metabolic syndrome [16].

In conclusion, we identified 22 *PPARD* variants, although potentially functional variants detected in the *PPARD* gene are rare. Synonymous variant c.489T>C (p.N163N; rs2076167) could be of clinical interest regarding its association with worse differentiated CRC. Ultimately, future studies in an independent clinical sample series will help to resolve whether any of the *PPARD* variants are potential modifiers of CRC susceptibility or prognosis.

## Supporting Information

Table S1 Primers used for PCR amplification and sequence analysis.
(DOCX)

Table S2 Rare *PPARD* variants in relation to the clinicopathological characteristics.
(DOCX)

Table S3 Homozygotic variant c.489C in relation to the clinicopathological characteristics.
(DOCX)
