# Supplementary material for: Variants of the PPARD Gene and Their Clinicopathological Significance in Colorectal Cancer
Source: PLoS One. 2013 Dec 31;8(12):e83952. doi: 10.1371/journal.pone.0083952 (PMC3877104; doi:10.1371/journal.pone.0083952)
Supplement: Table S1 — Primers used for PCR amplification and sequence analysis. (DOCX) [file pone.0083952.s001.docx]

**Table S1.** Primers used for PCR amplification and sequence analysis.

| **Exon** | **Primers 5'🡪 3'** | **Position of primers*^a^*** |
| --- | --- | --- |
|  | **(forward and reverse)** | **(length of PCR product)** |
| 4 | F*^b^*: AGGAGCAGGAGCAGAAGAAC | 73312-73748 |
|  | R: ACATGATGGCGTTTGCAGAG | (437 bp) |
| 5 | F: TGTTAAGTGGCTGAGGCGAG | 82473-82838 |
|  | R: TCTGCAATAGGTACAGTCAGG | (366 bp) |
| 6 | F: ACTTTCCTTCTCCATCTCCC | 84113-84533 |
|  | R: AGCCATGGTGCTTACTGTGC | (421 bp) |
| 7 | F: CATTCCTACCTTGCTGACTC | 86247-86812 |
|  | R: TCTGCCTGCCACAATGTCTC | (566 bp) |
| 8 | F: ACAATGCCTACCTGAAAAAC | 86512-87345 |
|  | R: TGCCAAGATCACAGGGACAC | (834 bp) |
| 9 | F: AGAAGTGGATTAAGACCAGG | 88090-88732 |
|  | R: GACAACAAAGACAGGAAGAG | (643 bp) |

*^a^*GenBank reference sequence NG_012345.1; *^b^*underlined primer was preferentially used for sequence analysis.
