# Supplementary material for: Variants of the PPARD Gene and Their Clinicopathological Significance in Colorectal Cancer
Source: PLoS One. 2013 Dec 31;8(12):e83952. doi: 10.1371/journal.pone.0083952 (PMC3877104; doi:10.1371/journal.pone.0083952)
Supplement: Table S2 — Rare PPARD variants in relation to the clinicopathological characteristics. (DOCX) [file pone.0083952.s002.docx]

**Table S2.** Rare *PPARD* variants in relation to the clinicopathological characteristics.

| Variant*^a^*, (exon/intron) | Group*^b^* | Male/ Female | Age (years) | Colon/ Rectum | Stage | Differentiation*^c^* |
| --- | --- | --- | --- | --- | --- | --- |
| c.1-101-3C>T, (i3) | I | F | 64 | R | III | poorly |
|  | I | F | 57 | R | I | moderately |
|  | II | F | 83 | R | I | - |
| c.89A>G, p.N30S, (ex4) + c.891C>T, p.I297I, (ex8) | I | F | 73 | C | II | well |
| c.424+31G>A, (i6) | **I** | **M** | **78** | **C** | **IV** | **moderately** |
| c.425-9C>T, (i6) | **I** | **M** | **50** | **C** | **II** | **poorly (Mu/Sig)** |
| c.425-44G>A, (i6) | I | M | 80 | R | III | moderately |
| c.542A>G, p.H181R, (ex7) | **I** | **M** | **68** | **R** | **III** | **poorly** |
| c.548A>G, p.Y183C, (ex7) | **I** | **F** | **35** | **C** | **II** | **moderately** |
| c.624G>A, p.T208T, (ex7) | I | F | 79 | C | II | poorly (Mu/Sig) |
| c.627+37G>A, (i7) | **II** | **F** | **60** | **C** | **III** | **-** |
| c.628-16G>A, (i7) | I | M | 66 | C | III | poor |
| c.1078+22G>A, (i8) | I | M | 65 | C | II | poorly (Mu/Sig) |
| c.1079-20C>T, (i8) | II | F | 51 | R | III | - |
| c.1326+122G>A, (ex9) | I | F | 78 | C | I | moderately |

***^a^***GenBank reference sequence are NG_012345.1 and NM_001171818.1:+1 corresponds to the A of the ATG translation initiation codon; *^b^* I – unselected CRC patients, II – sporadic patients, III – TCR patients, and IV – hereditary patients; ^c^Mu/Sig – mucinous differentiation or signet-ring cell carcinoma; novel variants are indicated in *bold*; sporadic variants are *underlined*.
