# Supplementary material for: Variants of the PPARD Gene and Their Clinicopathological Significance in Colorectal Cancer
Source: PLoS One. 2013 Dec 31;8(12):e83952. doi: 10.1371/journal.pone.0083952 (PMC3877104; doi:10.1371/journal.pone.0083952)
Supplement: Table S3 — Homozygotic variant c.489C in relation to the clinicopathological characteristics. (DOCX) [file pone.0083952.s003.docx]

**Table S3.** Homozygotic variant c.489C in relation to the clinicopathological characteristics.

| **Gender** | **Age** | **Location** | **Stage** | **Differentiation^a^** | **Survival** | **Censored^b^** |
| --- | --- | --- | --- | --- | --- | --- |
| **Male/Female** | **(year)** | **Colon/Rectum** |  |  | **(month)** |  |
| M | 88 | C | II | Mu/Sig | 16 | complete |
| M | 69 | C | IV | Poorly | 11 | complete |
| F | 74 | R | III | Mu/Sig | 44 | complete |
| M | 81 | C | III | Mu/Sig | 59 | complete |
| F | 87 | R | IV | Moderately | 2 | complete |
| F | 57 | C | II | Moderately | 96 | censored |
| F | 85 | C | III | Poorly | 0 | censored |
| F | 78 | R | II | Poorly | 87 | censored |
| F | 64 | R | III | Poorly | 28 | censored |
| F | 95 | C | II | Moderately | 36 | censored |
| F | 77 | C | II | Moderately | 65 | censored |

^a^Mu/Sig – mucinous differentiation or signet-ring cell carcinoma; ^b^complete – died of colorectal cancer, censored – stopped follow-up or death from other causes
